# A study of dyadic interdependence of control, social participation and occupation of adults who use long-term care services and their carers

**DOI:** 10.1007/s11136-017-1669-3

**Published:** 2017-08-07

**Authors:** Stacey Rand, Julien Forder, Juliette Malley

**Affiliations:** 1 Personal Social Services Research Unit (PSSRU), University of Kent, Cornwallis Building, Canterbury, CT2 7NF UK; 2 Personal Social Services Research Unit (PSSRU), London School of Economics and Political Science, Cowdray House, Houghton Street, London, WC2A 2AE UK

**Keywords:** Quality of life, Caregiver, Satisfaction with care, Long-term care, ASCOT, The actor-partner interdependence model

## Abstract

**Purpose:**

Unpaid care is an important source of support of people with long-term conditions. Interdependence of carers’ and care recipients’ quality of life would be expected due to the relational nature of caregiving. This study aims to explore interdependence of quality of life in carer/care-recipient dyads, especially in relation to mutual interdependence due to social feedback in the caregiving relationship and also the partner effects of one partner’s experience of long-term care support on the other’s outcomes.

**Methods:**

Using data collected in an interview survey of 264 adults with care support needs and their unpaid carers in England, we employed regression analysis to explore whether there is mutual interdependence of care-related quality of life within carer/care-recipient dyads for three quality of life attributes: *Control over daily life, Social participation* and *Occupation*. The influence of factors, including satisfaction with long-term care, were also considered on individuals’ and dyad partners’ care-related quality of life.

**Results:**

We found mutual interdependence of quality of life at the dyad-level for *Control over daily life*, but not *Occupation* or *Social participation*. A partner effect of care recipients’ satisfaction with long-term care on carers’ *Control over daily life* was also observed. Higher care recipient satisfaction with care services was associated with higher *Control over daily life*. By contrast, for *Social participation* and *Occupation*, there were only significant effects of care recipients’ satisfaction with long-term care and their own quality of life.

**Conclusions:**

These findings highlight the importance of considering the wider impact beyond the individual of long-term care on quality of life in the evaluation of long-term care policy and practice.

## Background

Unpaid care provided by family and friends of adults with long-term conditions is central to long-term care systems [1]. While the positive aspects of caregiving should be recognised [2–4], the experience of caregiving may negatively affect carers’ health, wellbeing and aspects of life: for example, carers may find it difficult to continue in employment [5–8] and to maintain relationships [9–11]. Policy-makers across Europe are at various stages of developing strategies to support carers in their caregiving role and to minimise the potential negative effects of caregiving on health and wellbeing [12].

In England, the carers’ strategy highlights that carers should be supported to balance caring with employment and access information about local services, benefits or other sources of support [13, 14]. Personalised support to meet the needs of carers and the people they support, as well as supporting carers to continue caring while maintaining their own health and wellbeing, is identified as an important aspect of policy strategy [14]. The Care Act (2014) considers the wellbeing of both care-recipients and carers and, notably, carers are entitled to formal long-term care support based on their own needs and specified outcomes.

The interest in carers’ and care-recipients’ wellbeing has emerged in a policy context in which care-related quality of life (CRQoL) measured by the Adult Social Care Outcomes Toolkit (ASCOT) and the ASCOT-Carer has been promoted as an overarching outcome indicator for long-term care services for both adults with support needs and their carers [15–17]. Care-related QoL is defined as aspects of quality of life, beyond health, that may be maintained or improved by long-term care services and are also valued by adults who use long-term care services or their carers [17–21]. The ASCOT measures were designed for the evaluation of long-term care policy and interventions [17, 21]. The construct of ASCOT CRQoL captures the broader impact of long-term care beyond health [17, 21–23]. The instrument has been recommended as a suitable instrument and also used for evaluation studies of long-term care interventions or policy and to evaluate the performance of long-term care systems [24–33].

These studies have focussed on the CRQoL of individual carers or care-recipients. Since caregiving occurs within the personal relationship between carer and care-recipient, however, it has been recognised that a narrow view of long-term care outcomes at the individual level may lead to an incomplete perspective [34, 35]. It is recognised that the outcomes of people in close relationships, such as caregiving relationships, are non-independent: that is, the outcome scores of two people in a close relationship are more similar to, or different from, one another than two people who are not members of the same pair or ‘dyad’ [36]. Non-independence may be attributed to correlated pairing of individuals (for example, a married couple may share a range of variables, like age or educational level that may have influenced their coming together as a pair) or shared contextual factors that influence both individuals after the dyad has formed (‘common fate’). In these cases, the outcomes of pairs are non-independent because they are affected by variables that are correlated at the dyad-level. Non-independence may also arise through *interdependence*, where the quality of life of one partner is directly affected by the other person. As such, interdependence is a subset of non-independence.

Care-related QoL is influenced by a set of observable characteristics such as age, sex, health, impairment and economic factors. By using dyadic analysis, we can differentiate the non-independence of these factors on outcomes due to correlated pairing or common fate from interdependence by incorporating partner effects (i.e. does the characteristic of one partner affect the other’s outcomes even after the same characteristic of the other partner is accounted for?). Furthermore, there may be *unobserved mutual* interdependence (for example, through mutual regard) that affects other-partner outcomes. A contribution of this paper is to account for these unobserved effects.

There have been studies of the non-independence of quality of life or wellbeing within caregiving dyads (for example, [37–45]). We are, however, aware of only one study of the non-independence of health-related QoL of carers and care-recipients that considers QoL as an outcome of care services [45]. This study explored the relationship between service satisfaction and patients’ and carers’ health-related QoL in the context of multidisciplinary stroke care services delivered at centres in the Netherlands [45]. We are not aware of studies of dyadic non-independence of care-related QoL outcomes in relation to community-based long-term care. This represents a gap in the evidence base, which could inform long-term care policy and practice that aims to support service users’ and their carers’ QoL by establishing the nature and extent of non-independence in relation to aspects of CRQoL [13, 14, 16]. Without the wider view of the non-independence of CRQoL within caregiving dyads, especially in terms of mutual interdependence, the impact of care services on QoL outcomes may be underestimated in economic evaluation of interventions or policy [46].

The primary aim of this study is, therefore, to test the hypothesis that there is mutual interdependence of the three CRQoL attributes that overlap between the ASCOT and ASCOT-Carer: *Control over daily life*; *Social participation*; and *Occupation* [17, 20] (see Table 1). These attributes are conceptualised as higher-order domains of care-related QoL that are more likely to be subject to mutual influence in the care-recipient and carer relationship than basic CRQoL attributes, like *Food and drink* (Netten et al. [17]), and two of these, *Control* and *Social,* are included in the Adult Social Care Outcomes Framework as key outcomes of long-term care in England [16]. A second aim was to investigate the hypothesis that mutual interdependence at the dyad-level would be more likely for *Control* than for the other two attributes and, more specifically, *unobserved* effects at the dyad-level would be stronger. This is due to the nature of close social relationships characterised by other regard, altruism and compromise that may affect an individual’s perceived ability to make choices about their everyday lives against the criteria of their own preferences. By contrast, it was anticipated that there would be a lesser degree of mutual interdependence at the dyad-level for *Social* and *Occupation* because the construct of *Social* and *Occupation* relates to social contact in general and activities completed alone or with others, respectively. Therefore, these CRQoL attributes would be expected to be less dependent on the sphere of mutual influence within the carer and care-recipient relationship than *Control*. Thirdly, whilst simultaneously testing and controlling for this hypothesised unobserved mutual interdependence of *Control, Social* and *Occupation* using dyadic data analysis [36], the study also explored interdependence in terms of partner effects of one individual’s characteristics on the dyad partner’s CRQoL. Specifically, we expected to observe partner effects of carers’ and care-recipients’ satisfaction with community-based care on the dyad partners’ CRQoL rating for each attribute.

**Table 1:** The ASCOT and ASCOT-Carer measures of care-related quality of life

Care-related QoL attribute	ASCOT Definition	ASCOT-Carer Definition
Control over daily life	The service user can choose what to do and when to do it, having control over his/her daily life and activities	The carer can choose what to do and when to do it, having control over his/her daily activities
Social participation and involvement	The service user is content with their social situation, where social situation is taken to mean the sustenance of meaningful relationships with friends and family, and feeling involved or part of a community should be important to the service user	The carer is content with their social situation, where social situation is taken to mean the sustenance of meaningful relationships with friends and family, and feeling involved or part of a community, should this be important to the carer
Occupation (‘doing things I value and enjoy’)	The service user is sufficiently occupied in a range of meaningful activities whether it be formal employment, unpaid work, caring for others or leisure activities	The carer is sufficiently occupied in a range of meaningful and enjoyable activities whether it be formal employment, unpaid work, caring for others or leisure activities
Personal safety	The service user feels safe and secure. This means being free from fear of abuse, falling or other physical harm and fear of being attacked or robbed	The carer feels safe and secure, where concerns about safety include fear of abuse, physical harm or accidents that may arise as a result of caring
Personal cleanliness and comfort	The service user feels he/she is personally clean and comfortable and looks presentable or, at best, is dressed and groomed in a way that reflects his/her personal preferences	N/A
Food and drink	The service user feels he/she has a nutritious, varied and culturally appropriate diet with enough food and drink he/she enjoys at regular and timely intervals	N/A
Accommodation cleanliness and comfort	The service user feels their home environment, including all the rooms, is clean and comfortable	N/A
Dignity	The negative and positive psychological impact of support and care on the service user’s personal sense of significance	N/A
Self-care	N/A	The carer feels that s/he is able to look after him/herself, in terms of eating well and getting enough sleep
Space and time to be yourself	N/A	The carer feels that s/he has enough space and time in everyday life to be him/herself away from the caring role and the responsibilities of caregiving
Feeling supported and encouraged	N/A	The carer feels encouraged and supported by professionals, care workers and others, in their role as a carer

By using a dyadic analytical approach, we are able to control for, and also test the interdependence of aspects of CRQoL in caregiving relationships. This approach reflects the long-term care policy focus on putting carers on an equal footing with care-recipients as co-clients whose needs and outcomes should also be recognised along with those of the care-recipient and also challenges the conceptualisation of carers as co-workers or resources to be utilised [47, 48]. Importantly, it also develops a broader view of long-term care outcomes beyond the individual that may be applied to the evaluation of the effectiveness of long-term care policy or interventions, without which the full impact of long-term care may be underestimated or misrepresented.

## Method

### Design

This cross-sectional study was conducted in 22 local authorities in England. These local authorities included metropolitan districts (*n* = 6), unitary authorities (*n* = 2), shire counties (*n* = 11) and London boroughs (*n* = 3) across the North-East, Yorkshire and the Humber (*n* = 3), North-West (*n* = 5), West Midlands (*n* = 2), South-West (*n* = 1), Eastern region (*n* = 3) and South-East or London (*n* = 8). Data were collected by face-to-face or telephone interview using a structured questionnaire completed by computer-assisted personal or telephone interviewing. The study design is reported in further detail elsewhere [49].

### Participants

A non-stratified random sample of eligible service users was identified by local authority staff from long-term care records held by the local authority. The sample was selected based on the following eligibility criteria: aged 18 years or older; in receipt of publicly funded community-based long-term care (e.g. home care, equipment, day centre); with a primary support reason of physical disability or sensory impairment, mental health or intellectual disability. The identified service users were sent a letter of invitation by the local authority and were asked to complete a return slip if they wished to participate. The fieldwork interviewers then contacted potential participants to discuss the research and arrange an interview.

The questionnaire included items from the social care module for people aged 65 or older to identify whether the respondent received unpaid help from family or friends with activities of daily living (ADLs) or instrumental ADLs (IADLs) [50]. If applicable, the care-recipient was asked to pass on a study information pack to the carer who was reported to spend the greatest number of hours per week on unpaid care. Of the 990 interviews completed with care-recipients, 739 respondents reported that they had unpaid help with I/ADLs. Of these, there were 510 cases (69.3%) where the respondent agreed to pass an invitation letter or the interviewer was able to speak directly with the carer. In total, 387 carer interviews were completed.

We excluded data collected from 71 people with an intellectual disability and their carers that were collected using an easy-read version of the questionnaire, which does not allow direct comparison in dyadic analyses. A further 18 cases, where someone other than the care-recipient answered the ASCOT, were also excluded. The analysis presented in this article was, therefore, conducted with data from 298 dyads, of which 233 (78.2%) received long-term care support for physical disability or sensory impairment and 65 (21.8%) for mental health needs.

### Data collection

Interviews were conducted by fieldwork interviewers between June 2013 and March 2014. Face-to-face interviews took place in a location convenient for the participant, typically at home. Carer interviews were conducted using the same mode of survey administration as for the care-recipient (face-to-face or telephone). All carer interviews were conducted within 60 days of the care-recipient interview, with 50.3% (*n* = 150) completed on the same day and 95.6% (*n* = 286) completed within three weeks. Written or verbal informed consent was obtained prior to all interviews.

### Questionnaire

The questionnaire included items from the Adult Social Care Survey (ASCS) [51, 52], the Survey of Adult Carers in England (SACE) [51, 53], the 2009/10 Survey of Carers in Households [54], and the social care questionnaire for people aged 65 or over [50]. Demographic data, including age and gender, were collected from all respondents. Items from the ASCS and SACE were used to collect information on self-rated overall health (five categories from very good to very bad), satisfaction with long-term care services (seven categories from extremely dissatisfied to extremely satisfied), and hours of unpaid care (seven categories, rated by the carer, from 0–4 to ≥100 h per week). All participants were rated their household financial situation (five categories from manage very well to severe difficulties) [54].

The fieldwork interviewer rated whether or not the carer was co-resident with the care recipient based on the contact details provided by participants. Ability to complete the 13 I/ADLs included in the social care questionnaire for people aged 65 or over was rated by care-recipients [50]. The number of I/ADLs where the respondent rated that they had difficulty to complete alone or needed help or were unable to complete alone were summed together into a scale from 0 to 13. The carers were also asked to rate the level of support from long-term care as: more than needed; about right; some more needed; or a lot more needed. This item was developed and piloted as an optional item for the SACE [53].

In addition to these items, the questionnaire included the ASCOT (care-recipient) and ASCOT-Carer (carer) measures of CRQoL [17–20]. These instruments have seven (ASCOT-Carer) or eight (ASCOT) attributes (see Table 1) that are rated as the ‘ideal state’ (3), ‘no needs’ (2), ‘some needs’ (1) or ‘high-level needs’ (0) (see Box 1). At the time of analysis, preference weights were available for ASCOT [17, 55], but not the ASCOT-Carer. Therefore, the equally weighted scores for both instruments were used in analyses.

**Table:** Box 1 ASCOT and ASCOT-Carer response levels

Response level	Description	Example: control over daily life^a^
Ideal state	The preferred situation, in which needs are met to the desired level	I have as much control over my daily life as I want
No needs	Where needs are met, but not to the desired level	I have adequate control over my daily life
Some needs	Where there are needs, but these do not have an immediate or longer-term health implication	I have some control over my daily life, but not enough
High-level needs	Where there are needs and these have an immediate or longer-term health implication	I have no control over my daily life

^a^Where control over daily life is defined as the choice to do things or have things done for you as you like and when you want

### Analysis

**Fig. 1:**
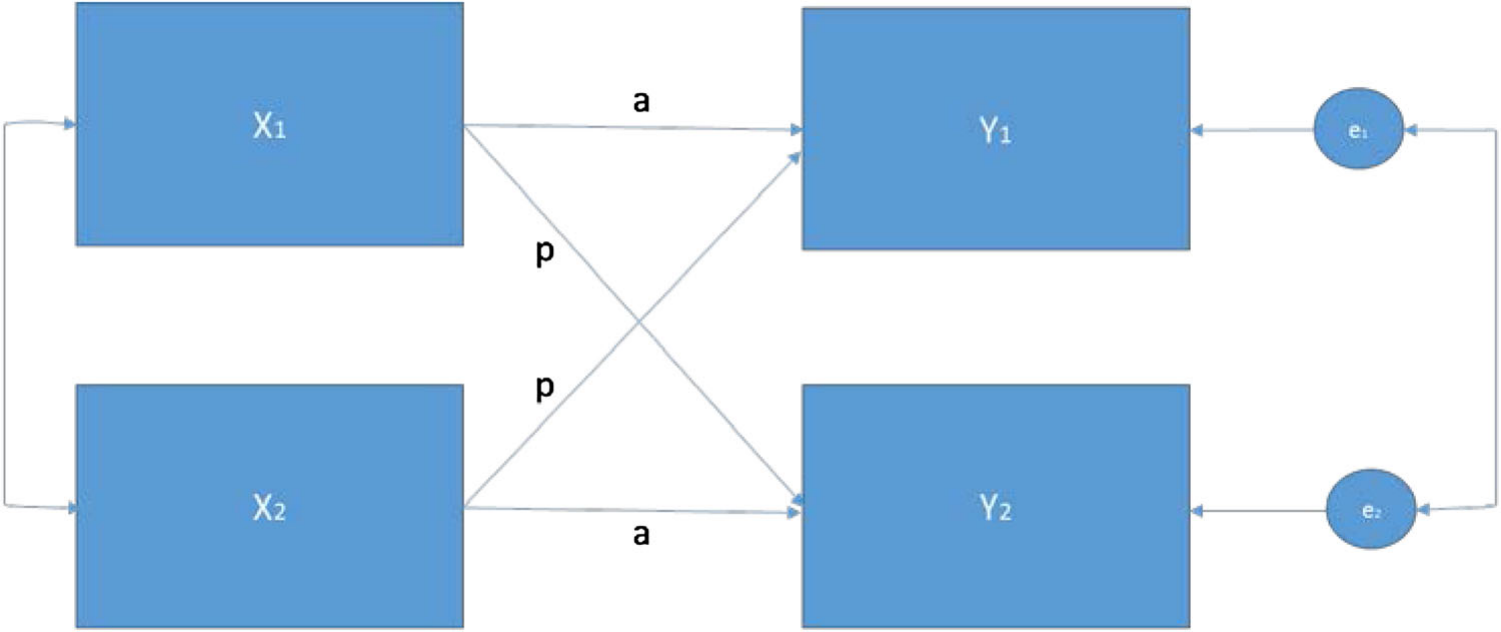
Actor-partner interdependence model (APIM)

To test the study hypotheses, we used the actor-partner interdependence model (APIM; see Fig. 1) [36]. The APIM takes into account, and also tests for, interdependence by considering actor and partner effects in the same analysis, as well as considering person-to-person and dyad-to-dyad variation [36]. The APIM enables consideration of both the effect of a variable on the individual’s and their dyad partner’s outcome simultaneously. Specifically, the *partner effects* in the APIM indicate an interpersonal effect (i.e. an association between an individual’s characteristics and the dyad partner’s outcome). *Actor effects* indicate an effect of an independent variable on the same individual’s dependent variable. In addition, both within- and between-dyad variation in independent variables were considered. The random effects (level 2) are taken to be an indicator of *unobserved mutual interdependence* specifically, although we cannot rule out that random effects could be caused by unobserved non-independence more generally.

**Table:** Box 2 Regression equation

The model can be written:	
$y_{ij} = \beta_{0} + \beta_{1} x_{ij} + \beta_{2} x_{ij}^{i} \beta_{3} x_{ij}^{ - i} + u_{j} + e_{ij}$	Where there are *j* = 1,…,*M* clusters (dyads) comprising two individuals, the care-recipient and the carer, denoted $i = 1,2$. The terms in the equation are
$y_{ij}$	The outcome variable score individual *i* in dyad *j*
$\beta_{1} x_{ij}$	The (fixed) effect on the outcome of a predictor variable $x_{ij}$ that is specific to the individual *i* in dyad *j* (and is not expected to have an effect on the outcome of the dyad partner *k* ≠ *i*). (e.g. the effect of survey administration by telephone on *Control over daily*)
$\beta_{2} x_{ij}^{i}$	The (fixed) effect of an individual’s predictor variable on the individual’s outcome variable (*actor effects*). (e.g. the effect of care-recipient age on the care-recipient’s *Control*)
$\beta_{3} x_{ij}^{ - i}$	The (fixed) effect of an individual’s predictor variable on the dyad partner’s (*k* = −*i*) outcome variable (*partner effects*). (e.g. the effect of carer age on the care-recipient’s *Control*, and vice versa)
$u_{j}$	A random effect which applies to the dyad *j*
$e_{ij}$	The error term

Three APIMs were calculated with the dependent variable of *Control, Social* and *Occupation* rated as the ideal state (3), no needs (2) or some needs/high-level needs (1). The APIMs were calculated as two-level multilevel mixed-effect ordered logistic regression using the two-intercept procedure for distinguishable dyads outlined in Kenny et al. [36, pp. 176–177]. In this model, the two levels of analysis are the individual (level 1, fixed effects) and dyad (level 2, random effects).^1^

The factors considered in the statistical models were selected based on the Production of Welfare model [56, 57], which has been used as a theoretical framework for exploring CRQoL [49, 58]. CRQoL is conceptualised as a function of various inputs that may be broadly summarised as: individual characteristics; environmental or contextual characteristics; underlying health condition(s); the effectiveness and intensity of long-term care; and other factors [49].

Based on this framework, the APIMs included actor and partner effects for sex, age, household finances, self-rated health, and satisfaction with services. Data collected only from carers (i.e. estimated hours of care per week, self-rated need for more formal support) were entered as actor fixed effects for carers and partner fixed effects for care-recipients. Conversely, variables collected only from care-recipients (i.e. I/ADLs) were entered as actor fixed effects for care-recipients and partner fixed effects for carers. Co-residence of the carer and care-recipient was modelled as an actor fixed effect for both carers and care-recipients. Dummy variables to distinguish carers from care-recipients and to indicate whether the interview was conducted by telephone or face-to-face were also included as fixed effects.

The type and intensity of long-term care received by the care-recipient and carer were also collected; however, these variables were not included in the models because of endogeneity. In England, long-term care services respond to maintain or improve a person’s QoL over time despite fluctuation in need. As such, the type and intensity of care is likely to be endogenous with CRQoL attributes of study.

The fixed effects generated from the multilevel mixed-effect ordered logistic regression may be interpreted as the output from an ordered logistic regression. The actor effects capture the effect of an individual characteristic on that individual’s outcome score, whilst controlling for the other fixed effects and also the random effect of the dyad. Likewise, the partner effects capture the effect of individual characteristics on the dyad partner’s outcome (e.g. the effect of care-recipients’ age on the carers’ *Control*).

Significant coefficients or odds ratios indicate an association between individual or service-related factors and CRQoL rating for the three attributes of study. The analysis tests the hypothesis that carers’ QoL would be more likely to be related to the care-recipient’s satisfaction with services than vice versa. If this hypothesis is not to be rejected, then we would expect to find significant partner effects of satisfaction with services on carers’ QoL for care-recipient’s satisfaction with services.

We modelled outcome effects to allow for a dyad-level random effect that is an unobserved effect that applies to each partner in the dyad, in order to capture any *unobserved* mutual influence effects that differentiate the effect of particular dyads on the outcome compared to other dyads.

A likelihood-ratio test, which compares the model to an ordered logistic regression, was applied to test whether the null hypothesis that the random effect equals zero could be rejected.

Due to the exclusion of 34 cases with one or more missing study variable, 264 dyads were considered in the three statistical models.

Analyses were conducted in Stata version 13 [59] using the *meologit* estimator.

## Results

### Descriptive statistics

The sample characteristics are shown in Table 2. The rating of the three overlapping ASCOT and ASCOT-Carer attributes are shown in Table 3. Spearman’s correlation was run to determine the relationship between carers’ and care-recipients’ ratings of *Control, Social* and *Occupation*. There were weak–moderate positive correlations for all three CRQoL attributes (*p* < 0.01). The correlation for *Control* is stronger (*rs* = 0.32, *p* < 0.01) than for Social (*rs* = 0.24, *p* < 0.01) or Occupation (*rs* = 0.23, *p* < 0.01). This indicates that the overall non-independence in carer and care-recipient dyads is larger for *Control* than *Social* or *Occupation*; however, to further explore the sources of non-independence and, more specifically, the non-independence due to *mutual interdependence,* we consider the results of the multilevel analysis.

**Table 2:** Descriptive statistics (*n* = 298 dyads)

	Care-recipient *n* (%) or mean (SD)	Carer *n* (%) or mean (SD)
Socio-demographics		
Sex: male	124 (41.6%)	137 (46.0%)
Age: ≥65 years	168 (56.4%)	135 (45.3%)
Ethnicity: white	271 (90.9%)^a^	272 (91.3%)
Household finances: alright, or some/severe difficulties	189 (63.4%)^a^	187 (62.8%)^a^
Health and disability		
Self-rated health: very good or good	94 (31.5%)	138 (46.3%)
Self-rated health: fair	111 (37.3%)	106 (35.6%)
Self-rated health: bad or very bad	93 (21.2%)	54 (18.1%)
Number of I/ADLs with difficulty	9.63 (3.42)	n/a
Community-based long-term care services		
Carer self-report that more formal support is needed	n/a	103 (34.6%)^a^
Extremely or very satisfied with services	143 (48.0%)	82 (27.5%)^a^
Caregiving situation		
Caring for ≥50 h per week	n/a	129 (43.3%)
Co-resident	n/a	223 (74.8%)
Survey administration		
Interview by telephone	45 (15.1%)	45 (15.1%)

^a^Missing values. Ethnicity: 3 (1.0%); household finances: 2 (0.6%); number of I/ADLs with difficulty: 17 (5.4%); carer self-report that more formal support is needed: 1 (0.3%); extremely or very satisfied with services: five care-recipients (1.7%) and eight carers (2.7%)

**Table 3:** Responses to the ASCOT and ASCOT-Carer (*n* = 298 dyads)

	ASCOT Care-recipient *n* (%)	ASCOT Carer *n* (%)	*Spearman’s Rho* (*p* value)
Control			
Ideal state	65 (21.8%)	75 (25.2%)	0.3193 (*p* < 0.001)
No needs	107 (35.9%)	114 (38.3%)	
Some needs	94 (31.5%)	100 (33.6%)	
High-level needs	32 (10.7%)	9 (3%)	
Occupation			
Ideal state	64 (21.5%)	65 (21.8%)	0.2262 (*p* < 0.001)
No needs	86 (28.9%)	88 (29.5%)	
Some needs	116 (38.9%)	123 (41.3%)	
High-level needs	31 (10.4%)	22 (7.4%)	
Social			
Ideal state	100 (33.6%)	108 (36.2%)	0.2427 (*p* < 0.001)
No needs	84 (28.2%)	90 (30.2%)	
Some needs	71 (23.8%)	74 (24.8%)	
High-level needs	43 (14.4%)	25 (8.4%)	
Missing values	0 (0%)	1 (0.3%)	

### Multilevel analysis

The results of the multilevel mixed-effects ordered logistic regressions are shown in Tables 4, 5, and 6.

**Table 4:** Multilevel ordered logistic regression: *Control*

	Odds ratio	Coeff. (B) (SE)
Fixed effects		
Actor effects: care-recipient		
Male	1.069	0.066 (0.342)
Aged 65+ years	0.625	−0.470 (0.347)
Household finances: alright, or some/severe difficulties	0.913	−0.092 (0.353)
Self-rated health: very good or good	2.024*	0.705 (0.334)
Co-resident with carer	0.399*	−0.918 (0.382)
Number of I/ADLs with difficulty	0.826**	−0.192 (0.049)
Satisfaction with services: extremely or very satisfied	1.444	0.367 (0.310)
Actor effects: carer		
Male	2.299*	0.832 (0.371)
Aged 65+ years	0.915	−0.088 (0.348)
Household finances: alright, or some/severe difficulties	0.863	−0.148 (0.353)
Self-rated health: very good or good	4.107**	1.413 (0.331)
Co-resident with care-recipient	0.395*	−0.930 (0.381)
Hours of unpaid caregiving per week: 50+ hours	0.380**	−0.967 (0.354)
Needs some or a lot more formal support	0.451*	−0.796 (0.333)
Satisfaction with services: extremely or very satisfied	1.222	0.201 (0.344)
Partner effects: on the care-recipient’s outcome		
Male	1.268	0.238 (0.354)
Aged 65+ years	1.059	0.057 (0.345)
Household finances: alright, or some/severe difficulties	0.472*	−0.751 (0.350)
Self-rated health: very good or good	0.714	−0.338 (0.321)
Hours of unpaid caregiving per week: 50+ hours	1.362	0.309 (0.353)
Needs some or a lot more formal support	0.320**	−1.139 (0.341)
Satisfaction with services: extremely or very satisfied	1.450	0.372 (0.341)
Partner effects: on the carer’s outcome		
Male	1.631	0.489 (0.360)
Aged 65+ years	1.785	0.579 (0.351)
Household finances: alright, or some/severe difficulties	0.796	−0.228 (0.354)
Self-rated health: very good or good	0.590	−0.528 (0.346)
Number of I/ADLs with difficulty	0.865**	−0.145 (0.048)
Satisfaction with services: extremely or very satisfied	1.919*	0.652 (0.317)
Interview by telephone	0.642	−0.443 (0.327)
Dyad member: carer	0.214	−1.541 (0.904)
Random effects		
Dyads	0.904	0.413
Number of dyads		264
Likelihood ratio test vs. ordered logit regression (*Χ* ^2^)		8.00**
Estimated cut-point 1 (κ1)	−3.706**	0.752
Estimated cut-point 2 (κ2)	−1.291	0.714

* *p* < 0.05, ** *p* < 0.01

**Table 5:** Multilevel ordered logistic regression: *Occupation (‘doing things I value and enjoy’)*

	Odds ratio	Coeff. (B) (SE)
Fixed effects		
Actor effects: care-recipient		
Male	0.852	−0.160 (0.301)
Aged 65+ years	1.274	0.242 (0.304)
Household finances: alright, or some/severe difficulties	0.726	−0.320 (0.315)
Self-rated health: very good or good	1.823*	0.600 (0.300)
Co-resident with carer	0.638	−0.450 (0.328)
Number of I/ADLs with difficulty	0.934	−0.068 (0.041)
Satisfaction with services: extremely or very satisfied	3.006**	1.101 (0.282)
Actor effects: carer		
Male	2.624**	0.965 (0.333)
Aged 65+ years	1.396	0.333 (0.314)
Household finances: alright, or some/severe difficulties	1.126	0.119 (0.324)
Self-rated health: very good or good	3.339**	1.206 (0.297)
Co-resident with care-recipient	0.341**	−1.075 (0.337)
Hours of unpaid caregiving per week: 50+ hours	0.408**	−0.898 (0.322)
Needs some or a lot more formal support	0.571	−0.560 (0.303)
Satisfaction with services: extremely or very satisfied	1.400	0.337 (0.303)
Partner effects: on the care-recipient’s outcome		
Male	0.839	−0.176 (0.313)
Aged 65 + years	1.827*	0.603 (0.306)
Household finances: alright, or some/severe difficulties	0.971	−0.030 (0.304)
Self-rated health: very good or good	1.067	0.065 (0.284)
Hours of unpaid caregiving per week: 50+ hours	0.915	−0.088 (0.309)
Needs some or a lot more formal support	0.841	−0.174 (0.293)
Satisfaction with services: extremely or very satisfied	0.878	−0.131 (0.307)
Partner effects: on the carer’s outcome		
Male	1.606	0.474 (0.322)
Aged 65+ years	1.119	0.112 (0.313)
Household finances: alright, or some/severe difficulties	1.196	0.179 (0.321)
Self-rated health: very good or good	0.881	−0.126 (0.311)
Number of I/ADLs with difficulty	0.922	−0.081 (0.041)
Satisfaction with services: extremely or very satisfied	1.002	0.002 (0.286)
Interview by telephone	0.932	−0.070 (0.280)
Dyad member: carer	1.175	0.162 (0.871)
Random effects		
Dyads	0.158	0.276
Number of dyads		264
Likelihood ratio test vs. ordered logit regression (*Χ* ^2^)		0.37
Estimated cut-point 1 (κ1)	−0.431	0.644
Estimated cut-point 2 (κ2)	1.315*	0.647

* *p* < 0.05, ** *p* < 0.01

**Table 6:** Multilevel ordered logistic regression: *Social and involvement*

	Odds ratio	Coeff. (B) (SE)
Fixed effects		
Actor effects: care-recipient		
Male	1.073	0.070 (0.317)
Aged 65+ years	1.764	0.568 (0.313)
Household finances: alright, or some/severe difficulties	0.563	−0.575 (0.324)
Self-rated health: very good or good	1.852*	0.616 (0.306)
Co-resident with carer	1.001	0.001 (0.354)
Number of I/ADLs with difficulty	0.878**	−0.131 (0.045)
Satisfaction with services: extremely or very satisfied	4.092**	1.409 (0.297)
Actor effects: carer		
Male	2.307*	0.836 (0.345)
Aged 65 + years	0.819	−0.199 (0.311)
Household finances: alright, or some/severe difficulties	0.952	−0.049 (0.319)
Self-rated health: very good or good	2.205**	0.791 (0.290)
Co-resident with care-recipient	0.607	−0.500 (0.356)
Hours of unpaid caregiving per week: 50+ hours	0.524*	−0.646 (0.312)
Needs some or a lot more formal support	0.492*	−0.709 (0.294)
Satisfaction with services: extremely or very satisfied	1.399	0.336 (0.315)
Partner effects: on the care-recipient’s outcome		
Male	0.941	−0.061 (0.329)
Aged 65+ years	1.279	0.246 (0.312)
Household finances: alright, or some/severe difficulties	0.934	−0.068 (0.323)
Self-rated health: very good or good	1.494	0.401 (0.287)
Hours of unpaid caregiving per week: 50+ hours	1.285	0.251 (0.312)
Needs some or a lot more formal support	0.738	−0.303 (0.299)
Satisfaction with services: extremely or very satisfied	0.668	−0.404 (0.318)
Partner effects: on the carer’s outcome		
Male	1.489	0.398 (0.329)
Aged 65+ years	2.170*	0.775 (0.317)
Household finances: alright, or some/severe difficulties	1.159	0.148 (0.324)
Self-rated health: very good or good	1.531	0.426 (0.316)
Number of I/ADLs with difficulty	0.897*	−0.108 (0.044)
Satisfaction with services: extremely or very satisfied	1.396	0.333 (0.286)
Interview by telephone	0.776	−0.253 (0.292)
Dyad member: carer	1.443	0.367 (0.876)
Random effects		
Dyads	0.340	0.301
Number of dyads		264
Likelihood ratio test vs. ordered logit regression (*Χ* ^2^)		1.63
Estimated cut-point 1 (κ1)	−0.968	0.669
Estimated cut-point 2 (κ2)	0.691	0.668

* *p* < 0.05, ** *p* < 0.01

The likelihood ratio test was significant for the analysis with *Control* as the outcome variable, but not *Social* (*p* = 0.10) or *Occupation* (*p* = 0.27), which shows that there is a significant random effect at the dyad level, an indicator of mutual interdependence from unobserved factors, for *Control,* but not for *Social* or *Occupation.*

### Control

The results of the analysis for *Control* are shown in Table 4. Care-recipients who live with their carer or report difficulty with a greater number of I/ADLs are significantly less likely to report a high level of *Control* at the 5% level. Care-recipients who report good or very good health are significantly more likely to report higher *Control.* When looking at the relationship between carers’ characteristics and their own rating of *Control,* those who reported good self-reported health or are male were more likely to have higher *Control.* There were also significant associations between worse rating of *Control* by carers and co-residence with the care-recipient, high-intensity caregiving (≥50 h/week), and carers’ perception that they needed some or a lot more long-term care support. Interestingly, the actor effects of satisfaction with services for both carers and care-recipients were not significant at the 5% level.

Four partner effects, which indicate mutual interdependence within the dyad through a relationship between one individual’s characteristics and the other dyad member’s outcome, were found to be significant at the 5% level. The care-recipient’s rating for *Control* was negatively associated with the carer’s rating of household financial difficulties and also report by the carer that they felt they needed more formal support. The number of I/ADLs with difficulty or unable to complete alone reported by the care-recipient, which is an indicator of care-recipients’ long-term care needs, was negatively associated with carers’ rating of *Control*. The rating of *Control* by carers at the ideal state or no needs was significantly positively associated with care-recipient satisfaction with long-term care support.

After controlling for other fixed effects, the difference in carer and care-recipient *Control* did not reach significance.

### Occupation

The results of the regression analysis with *Occupation* are shown in Table 5. There were two significant actor effects for care-recipients. Self-rated good health and satisfaction with long-term care support were positively associated with better scores of *Occupation* by care-recipients. There were four significant actor effects estimated for carers. Male carers and those who reported good health were more likely to report a higher outcome state for *Occupation*. High-intensity informal caregiving of 50 or more hours per week, as well as the carers’ perception that they needed more formal support, were associated with lower rating of *Occupation*.

Only one partner effect was significant at the 5% level. Care-recipients with a carer aged 65 years or older were more likely to report a better outcome state for *Occupation*. There was also a trend towards significance (*p* = 0.051) for the association between a higher level of long-term care need reported by the care-recipient (i.e. the number of I/ADLs with difficulty) and lower rating of *Occupation* by carers.

The dummy variable to capture differences between care-recipients and carers, whilst controlling for other factors, was not significant at the 5% level. This indicates that there is no significant difference in rating of *Occupation* between carers and care-recipients.

### Social

The results for *Social* are shown in Table 6. Care-recipients who reported good self-rated health and satisfaction with services were more likely to rate higher QoL in this attribute. A significant negative association was found between care-recipients’ rating of *Social* and higher number of I/ADLs with difficulty. Male carers and those who reported good self-rated health were more likely to report a higher outcome state for *Social*. Carers who provided 50 or more hours of unpaid care per week or reported that they needed more formal support were less likely to rate good QoL in this attribute.

Two partner effects on carers’ *Social* were significant at the 5% level. First, carers whose care-recipients were aged 65 years or older were more likely to rate higher *Social*. Second, a higher number of I/ADLs with difficulty rated by care-recipients is significantly negatively associated with carer QoL in this attribute.

After controlling for other fixed effects, the variable to distinguish carers and care-recipients did not reach significance at the 5% level.

## Discussion

The aim of this study was to explore the non-independence of three care-related QoL attributes (*Control over daily life, Social* and *Occupation)* within the caregiving relationship with a particular focus on the contribution of mutual interdependence at the dyad-level to non-independence overall. To the best of our knowledge, this is the first study to use the APIM to explore nature and type of dyadic non-independence of *Control over daily life, Social* and *Occupation* in the context of community-based long-term care. This study used the APIM to simultaneously explore and also control for the effects of individual and contextual factors on each of the three CRQoL attributes while testing for mutual interdependence within the caregiving relationship at the dyad-level.

Interdependence may be *observed directly* as the effect of individual characteristics on the QoL of the individual’s partner in dyad (partner effects). It can also arise from *unobserved* effects that differentiate the QoL of both partners in a dyad from the QoL of partners in other dyads (*unobserved* mutual interdependence at the dyad-level). Of the three care-related QoL attributes considered in this study, there was only evidence for *unobserved* mutual interdependence at the dyad-level for *Control*. This *unobserved* mutual interdependence is an indicator of the mutual influence of one person’s perception of their control over daily life on another’s through social interaction within the caregiving relationship. This finding is consistent with evidence from qualitative studies that carers frequently experience a loss of autonomy due to the shared experience of restrictions created by the care-recipient’s needs and powerlessness in navigating the long-term care system [60]. The restrictions of caregiving on their lifestyle and future plans may also contribute to carers’ perceptions of a loss of control over their everyday lives [10], especially if the cared-for person has a health condition with an uncertain prognosis or if the carer prioritises care-recipient’s needs over their own [9, 60–62].

This study provides evidence that carers’ and care-recipients’ rating of *Control over daily life* are mutually interdependent, which affirms the place of choice and control in carers’ policy strategy in England [14, 63] and the importance of considering the needs and outcomes of care-recipients and carers together in long-term care policy and practice: for example, policies that just focus on care-recipients (as is often the case), should also account for the ‘collateral’ effects on the outcomes of the other partner.

Control, choice and independence for carers are central to the personalisation agenda in long-term care services [64]; however, the policy aim to place carers on an equal footing with care-recipients has often focussed narrowly on choice in relation to the use of long-term care services rather than in terms of a broader construct that also captures choice over whether or not to care, which care tasks to undertake, and decisions related to everyday life (e.g. whether to combine care and paid employment) [65, 66].

While carers’ choice whether to care is affirmed in policy [14, 63], this is often not translated into practice because the exercise of choice by carers is problematic in long-term care systems that rely on the unpaid support they provide to adults with support needs [65]. This paradoxically locates carers as co-workers in the provision of care, whose outcomes may be subordinate to the needs and outcomes of care-recipients, and also co-clients in the use of long-term care services, whose outcomes are equally important [47]. While a view of outcomes beyond the individual may not resolve the problematic position of carers within long-term care policy in England, an increased awareness of mutual interdependence in quality of life, especially in relation to *Control*, may reinforce the wider focus on people with support needs and also their carers as co-clients of long-term care services. It also provides a way of capturing the wider impact of long-term care, so that the effects are not misrepresented or underestimated in the evaluation of interventions or policy.

This study was also concerned with the *observed* sources of interdependence or ‘partner effects’. Partner effects were observed for all three CRQoL attributes of study. As would be expected, a higher level of care-recipient long-term care need (number of I/ADLs with difficulty) was related to lower carer rating of *Control* and *Social*. This is consistent with studies that have found that carers’ experience and QoL are influenced by the needs of the care-recipient either directly or indirectly through engagement with different types of caregiving tasks [5, 67, 68]. Partner effects were also observed for other individual and contextual characteristics: for example, the age of the carer on the care-recipient’s rating of *Occupation* and, conversely, between the care-recipient’s age and carer rating of *Social*. Interestingly, significant partner effects were only observed for the variables related to the context and long-term care for *Control.* Specifically, carer-report of the need for more support and difficulty with household finances were significantly associated with lower rating of *Control* by care-recipients. Also, the care-recipient’s satisfaction with long-term care support was significantly associated with carers’ rating of higher *Control*. This is consistent with qualitative evidence that carers’ perception of their QoL is influenced by their view of how satisfied the care-recipient is with long-term care support, with carers who perceive that the care-recipient is satisfied with long-term care support more able to rely on care services, thus improving their own sense of *Control over daily life* [47].

While the analysis presented in this paper contributes to the existing literature by exploring non-independence within caregiving dyads in the context of community-based long-term care in England, the results also contribute to the literature on the relationship between individual and contextual characteristics and care-related QoL (actor effects) [30, 31]. The findings were broadly consistent with this literature, as well as other studies of QoL in relation to caring or long-term care: for example, there were also significant relationships between high intensity caregiving and lower QoL for *Control, Social* and *Occupation,* which is consistent with other studies [5, 9, 69]. Likewise, male carers were found to report higher QoL than female carers, which is again consistent with other studies that have found lower levels of emotional wellbeing and higher levels of depression in female carers [70–73]. Co-residence was related to lower carer and care-recipient *Control*, as well as lower rating of *Occupation* by carers, which is in line with studies that have found carers who live with the care-recipient are more involved in caregiving tasks and experience greater role captivity [11].

Consistent with the production of welfare framework and other studies of long-term care outcomes [31, 49, 56, 57], individual characteristics related to health status and long-term care needs were also found to be related to outcomes. Specifically, self-rated health was associated with both carers’ and care-recipients’ own rating of *Control, Social* and *Occupation*; care-recipients with greater long-term care needs had lower ratings of *Control* and *Social*; and carers who reported that they and the care-recipient needed more long-term care support had lower ratings for *Control* and *Social*. Furthermore, in addition to the partner effect of satisfaction with services and *Control* discussed before, there were significant actor effects of care-recipient satisfaction with services and rating of *Occupation* and *Social*. These findings contribute to knowledge of the factors associated with QoL outcomes in the context of long-term, which may be used to inform policy and practice, for example, in the identification of ‘at risk’ groups who may benefit from targeted support.

This study has some limitations. First, our study is limited to the context of caregiving relationships in England, in which the care-recipient uses publicly-funded long-term care support. Second, while the ASCOT instruments have been adapted to facilitate data collection, for example by easy-read format or mixed-methods [74, 75], the data collected in this study only used self-report and, thus, excluded adults with cognitive or communication impairments who were unable to complete the standard version of the tool as an interview.

## Conclusion

These findings highlight the importance of considering the wider impact of long-term care beyond individual care-recipients. This analysis provides evidence for mutual interdependence from unobserved factors in the rating of *Control* in the caregiving relationship, as well as observed interdependence in terms of partner effects for *Control, Social* and *Occupation*. If long-term care policy and practice aims to improve the QoL of care-recipients and also carers on an equal footing with care-recipients, then there should be consideration of the wider effect of long-term care beyond individuals and also the influence of the caregiving relationship on CRQoL outcomes.

## Footnotes

1 The dataset was structured such that each case represents an individual carer or care-recipient. Individuals were nested within dyads identified by a unique dyad code. Dyad members were also distinguishable using a dummy variable coded as care-recipient (0) or carer (1). The models included actor (within) and partner (between) fixed effects for both carers and care-recipients. The actor effects were captured using variables coded as zero (0) for the partner: for example, the actor variable for carers’ age was coded as zero (0) for carers aged 18–64 years, one (1) for carers aged 65 years or over, and zero (0) for all care-recipients. The partner effects were captured using variables coded as zero (0) for the actor: for example, the partner variable for carers age was coded as zero (0) for care-recipients whose carer was aged 18–64 years, one (1) for care-recipients who carer was aged 65 years or over, and zero (0) for all carers.
